# Green material selection for sustainability: A hybrid MCDM approach

**DOI:** 10.1371/journal.pone.0177578

**Published:** 2017-05-12

**Authors:** Honghao Zhang, Yong Peng, Guangdong Tian, Danqi Wang, Pengpeng Xie

**Affiliations:** 1 Key Laboratory of Traffic Safety on Track of Ministry of Education, School of Traffic and Transportation Engineering, Central South University, Changsha, China; 2 Transportation College, Jilin University, Changchun, China; 3 College of Automotive Engineering, Jilin University, Changchun, China; Southwest University, CHINA

## Abstract

Green material selection is a crucial step for the material industry to comprehensively improve material properties and promote sustainable development. However, because of the subjectivity and conflicting evaluation criteria in its process, green material selection, as a multi-criteria decision making (MCDM) problem, has been a widespread concern to the relevant experts. Thus, this study proposes a hybrid MCDM approach that combines decision making and evaluation laboratory (DEMATEL), analytical network process (ANP), grey relational analysis (GRA) and technique for order performance by similarity to ideal solution (TOPSIS) to select the optimal green material for sustainability based on the product's needs. A nonlinear programming model with constraints was proposed to obtain the integrated closeness index. Subsequently, an empirical application of rubbish bins was used to illustrate the proposed method. In addition, a sensitivity analysis and a comparison with existing methods were employed to validate the accuracy and stability of the obtained final results. We found that this method provides a more accurate and effective decision support tool for alternative evaluation or strategy selection.

## Introduction

While rapid urbanization and industrialization exacerbate the rate of resource shortages and environmental pollution worldwide, the promotion of sustainability has been increasing and gaining public momentum [1–3]. In the summit of the Group of Twenty (G20), sustainability in the global economy was one of the most crucial issues that have become highly appreciated. Subsequently, collective action plans regarding the sustainable development agenda in 2030 were formulated by the G20 leaders. Moreover, a large body of literature and research studies on sustainability, particularly involving material selection, have been generated by the relevant experts in recent years [4–7].

Green material selection, also called sustainable material selection, plays a significant role throughout the design-manufacturing process, which seeks to guarantee product performance and reduce the entire life-cycle impact to the environment and human health. Thus, it has been the subject of many studies [8–10]. Akadiri *et al*. [11] presented a novel model for building material selection via the fuzzy extended analytical hierarchy process (AHP) techniques. Maniya and Bhatt [12] applied the preference selection index method to seek a proper material that meets the design engineers' requirements. Chatterjee *et al*. [13] explored a new integrated multi-criteria decision making (MCDM) method that combines the complex proportional assessment method and the evaluation of mixed data method to select the optimal material alternative. However, when selecting the suitable green material for design products, various criteria or attributes, e.g., cost, physical property and environmental performance, should be considered simultaneously rather than only considering a single criterion for design engineers. Obviously, each material has distinctive performance for different properties, and no one can satisfy all the relevant properties. Therefore, green material selection should be viewed as a complex MCDM problem. To address this problem, a systematic and reasonable method is required.

Up to now, there are two types of methods to solve MCDM problem: synthetical assessment approaches[14–24], e.g., multi-attribute utility theory, technique for order performance by similarity to ideal solution (TOPSIS) and approaches based on the theory of life cycle assessment [25]. In addition, some integrated methods have been successfully applied to overcome the shortcomings of single one [26–29], e.g., AHP and TOPSIS, AHP and vlse kriterijumska optimizacija kompromisno resenje (VIKOR). However, this existing decision analysis approaches still have some problems to be solved in the decision model [30]. Therefore, to obtain the optimal material more reasonably and reduce subjectivity, it is essential to promote and optimize the assessment process.

This study proposes a hybrid MCDM approach combining DANP and G-TOPSIS that integrated GRA with TOPSIS to select optimal green material for sustainability based on product's needs. Among them, DEMATEL is used to analyze the interrelationship and influence of each criterion, and ANP is employed to calculate the final weights of criteria and evaluate each alternative to select the optimal green material via G-TOPSIS. An empirical application of rubbish bins is used to illustrate this hybrid MCDM method. In addition, a sensitivity analysis and comparison with existing methods are employed to validate the accuracy and stability of the obtained final results. In comparison with existing studies, this work has the following three distinctive contributions: 1) Establish a suitable hierarchy structure of each criterion, considering the economic, environment and physical properties. 2) Propose a hybrid MCDM approach that combines DANP and G-TOPSIS to obtain the weight of each criterion and select the optimal green material logically and effectively. 3) To reduce/avoid subjectivity and irrationality, a nonlinear programming is applied to make G-TOPSIS more reasonable.

The remainder of this paper is organized as follows. Section 2 describes the literature review. The hybrid MCDM method is proposed in Section 3. In Section 4, the proposed method is presented and applied to the case of rubbish bins. Analysis and discussion of the results are presented in Section 5. In last section, the conclusions are presented.

## Literature review

Material selection has great importance in the design and development of products, and it is also critical for the success and competitiveness of the producers. Improper selection of materials may result in damage or failure of an assembly and significantly decreases the performance of products, thus negatively affecting productivity, profitability and reputation of an organization [31–32]. In the literature, many investigations and studies have been conducted in various contexts to select material alternative based on different requirements/backgrounds which include eco-innovation, green manufacturing, market demand and so on, for real engineering processes [16, 33–36]. Note that sustainability as a philosophy has been gradually entering design and manufacturing industries for products to combat climate change under umbrella terms [37–39]. It is essential to carry out the study of material selection under the background of sustainable development [40–41].

The selection of material alternatives is a multi-objective problem subject to compound constraints which can be viewed as a complex MCDM problem [23]. The objectives and criteria in the material selection process are often in conflicts and it involves trade-offs amongst decisive criteria. To ease out the material selection procedure and make the right decision, a systematic and efficient approach is required. Nowadays, a lot of mathematical techniques have been developed and applied in material selection field. For example, Anojkumar *et al*. [42] developed a hybrid MCDM method by combing four MCDM methods for solving pipes material selection difficulty in sugar industry to choose the best pipe material. Liu *et al*. [31] presented an interval 2-tuple linguistic VIKOR (ITL-VIKOR) method for solving the material selection problem under uncertain and incomplete information environment. Huang *et al*. [43] presented a new MCDM model and uncertainty analysis method for the environmentally conscious material selection problem. TOPSIS method is employed and uncertainty analyses were performed for model flexibility and efficiency by addressing the materials selection challenge. Liu *et al*. [44] proposed a hybrid decision making approach integrating induced aggregation operators into VIKOR in an MCDM problem regarding the selection of materials and the results are compared for different types of standardized distance aggregation operators. Table 1 presents the commonly used approaches in material alternatives evaluation and selection.

**Table 1:** Applications of MADM methods for material selection in different areas.

Type	Method	Author(s)	Problem
MCDM approaches	AHP	Desai *et al*. [45]	Material selection in product design
	TOPSIS	Rahman *et al*. [46]	A decision support system for optimal roofing material selection
	VIKOR	Prasenjit *et al*. [9]	Material selection application
	GRA	Zhao *et al*. [41]	Commercially available materials selection in sustainable design
Hybrid MCDM approaches	AHP and TOPSIS	Kumar and Singal [34]	Penstock material selection in small hydropower plants
		Rao *et al*. [16]	Material selection for a given engineering design
		Anojkumar *et al*. [47]	Material selection in sugar industry
	ANP and TOPSIS	Onut *et al*. [48]	Selection of the suitable material handling equipment
	TOPSIS and DOE	Tansel Ic [49]	Robot selection problem
	TOPSIS and VIKOR	Shanian and Savadogo [50]	Selection of mass produced non-heat-treatable cylindrical cover material.
	DANP and VIKOR	Hsu *et al*. [51]	The best vendor selection for conducting the recycled material
		Liu *et al*. [52]	Material selection with target-based criteria
	Finite element analysis and ELECTRE	Shanian *et al*. [53]	Materials selection of gas turbine components
MCDM approaches with uncertain theory	Fuzzy TOPSIS	Maity and Chakraborty [54]	Grinding wheel abrasive material selection
		Mayyas *et al*. [55]	Eco-material selection
	Fuzzy ANP and PROMETHEE	Tuzkaya *et al*. [56]	Material handling equipment selection problem
	Fuzzy AHP and VIKOR	Anojkumar *et al*. [42]	Pipe material selection in sugar industry
	Interval 2-tuple linguistic VIKOR	Liu *et al*. [31]	Material selection for an engineering design
	Fuzzy VIKOR	Girubha *et al*. [57]	Material selection of an automotive component
	Fuzzy extended AHP	Akadiri *et al*. [11]	Sustainable materials selection for building projects
	Fuzzy AHP and TOPSIS	Anojkumar *et al*. [42]	Pipe material selection in sugar industry
		Aly *et al*. [58]	Best design concept and material selection process
		Rathod *et al*. [59]	Phase change material selection
	Fuzzy AHP, VIKOR and TOPSIS	Anojkumar *et al*. [60]	Material selection in sugar industry

The literature review demonstrates that the majority of researchers concentrated on material selection methods applying MCDM approaches. Although the existing methods provide many useful tools for material selection. However, most of them still ignore some aspects, e.g., physical properties is rarely considered which plays an significant role in the assessment process for green material alternatives; TOPSIS method as a commonly used tool is not suitable to assess all kinds of material alternatives due to its measurement scale is distance. Therefore, this study proposes a hybrid MCDM approach combining DANP and G-TOPSIS that integrated GRA with TOPSIS to select optimal green material for sustainability based on product's needs and formulate a new hierarchical structure including economic, environment, and physical properties.

## Solution methodology

A hybrid MCDM approach that combines DANP and G-TOPSIS is proposed to select the optimal green material alternative for certain product. DANP is applied to analyze the influences and interrelationships among each criterion and obtain the final weights of each criterion. The optimal alternative will be evaluated via G-TOPSIS. The specific procedures and processes of both phases are summarized in the following sub-sections.

### DANP

ANP, as an extension of AHP, was proposed by Saaty to address the interdependence and feedback among each criterion and alternative in the practical problem [61, 62]. However, the normalization method of supermatrix is not appropriate because each cluster, which originates from the allocation of each criterion in a column, has the same weight in the traditional process. There are varying degrees of impact among the clusters of criteria in the practical problem [29, 63]. Thus, DEMATEL is applied to improve the normalization process in ANP, namely DANP. It has been successfully employed in various fields, e.g., vendor selection and material selection [6, 64–65]. The procedure can be summarized as shown in Appendix A.

### G-TOPSIS

TOPSIS is an MCDM method proposed by Hwang and Yoon in 1981 [66]. The operating principium is that the optimal solution must satisfy certain conditions, i.e., the shortest distance from the positive-ideal solution and the longest distance from the negative-ideal solution [67, 68]. It has been commonly applied in various fields, e.g., weapon selection, material selection, and alternative evaluation [69–72]. However, some problems cannot be resolved by TOPSIS in several special cases, e.g., when the distances of the alternatives to positive-ideal and negative-ideal solutions is equal. Thus, G-TOPSIS, which integrates GRA with TOPSIS, is proposed to obtain the final rank of each alternative and select the optimal green material for certain product. Additionally, to avoid the subjectivity and irrationality, a nonlinear programming model with constraints is proposed to obtain the integrated closeness index based on the similarity closeness index from GRA and the distance closeness index from TOPSIS. The procedure can be summarized as follows:

**Step 1:** Construct a decision matrix for the selection of the optimal material. The decision matrix *X* = [*x*_*ij*_]_*n*×*m*_ can be presented as Eq (1).
  $$X=\begin{array}{cc} & \begin{array}{ccccc} B_{1} & \cdots & B_{j} & \cdots & B_{m} \end{array}\\ \begin{array}{c} A_{1}\\ \vdots \\ A_{i}\\ \vdots \\ A_{n} \end{array} & \left[\begin{array}{ccccc} x_{11} & \cdots & x_{1j} & \cdots & x_{1m}\\ \vdots & \ddots & \vdots & \ddots & \vdots \\ x_{i1} & \cdots & x_{ij} & \cdots & x_{im}\\ \vdots & \ddots & \vdots & \ddots & \vdots \\ x_{n1} & \cdots & x_{nj} & \cdots & x_{nm} \end{array}\right] \end{array}$$ (1)
  where *x*_*ij*_ is a crisp value that indicates the performance rating of each alternative *A*_*i*_ (*i* = 1, 2, …, *n*) with respect to each criterion *B*_*j*_ (*j* = 1, 2, …, *m*).
**Step 2:** Obtain the normalized decision matrix *Z* combined with the weight vector of criteria ***ω*** that is obtained via DANP.
  For the benefit criteria, the normalized value *y*_*ij*_ could be calculated as
  $$y_{ij}=\frac{x_{ij}}{\underset{i}{\text{max}}\;x_{ij}},\left(i=1,2,\cdots ,n;j=1,2,\ldots ,m\right)$$ (2)
  For the cost criteria, the normalized value *y*_*ij*_ could be calculated as
  $$y_{ij}=\frac{\underset{i}{\text{min}}\;x_{ij}}{x_{ij}},\left(i=1,2,\cdots ,n;j=1,2,\ldots ,m\right)$$ (3)
  $$Z=\omega^{T}Y=\begin{array}{cc} & \begin{array}{ccccc} B_{1} & \cdots & B_{j} & \cdots & B_{m} \end{array}\\ \begin{array}{c} A_{1}\\ \vdots \\ A_{i}\\ \vdots \\ A_{n} \end{array} & \left[\begin{array}{ccccc} \omega_{1}^{\prime }y_{11} & \cdots & \omega_{j}^{\prime }y_{1j} & \cdots & \omega_{m}^{\prime }y_{1m}\\ \vdots & \ddots & \vdots & \ddots & \vdots \\ \omega_{1}^{\prime }y_{i1} & \cdots & \omega_{j}^{\prime }y_{ij} & \cdots & \omega_{m}^{\prime }y_{im}\\ \vdots & \ddots & \vdots & \ddots & \vdots \\ \omega_{1}^{\prime }y_{n1} & \cdots & \omega_{j}^{\prime }y_{nj} & \cdots & \omega_{m}^{\prime }y_{nm} \end{array}\right] \end{array}$$ (4)
**Step 3:** Establish the positive-ideal and negative-ideal solutions. Based on the operating principium of TOPSIS, the positive-ideal and negative-ideal solutions play a significant role in the arithmetic process and can be obtained as Eqs (5) and (6).
  $$Z_{\text{j}}^{+}=\left\{\underset{1\leq i\leq n}{\text{max}}\left({\left\{z_{ij}\right\}}_{i=1}^{n}\right)|j\in J^{+},\underset{1\leq i\leq n}{\text{min}}\left({\left\{z_{ij}\right\}}_{i=1}^{n}\right)|j\in J_{-}\right\}=\left(z_{1}^{+},z_{2}^{+},\ldots ,z_{m}^{+}\right)$$ (5)
  $$Z_{\text{j}}^{-}=\left\{\underset{1\leq i\leq n}{\text{min}}\left({\left\{z_{ij}\right\}}_{i=1}^{n}\right)|j\in J^{+},\underset{1\leq i\leq n}{\text{max}}\left({\left\{z_{ij}\right\}}_{i=1}^{n}\right)|j\in J_{-}\right\}=\left(z_{1}^{-},z_{2}^{-},\ldots ,z_{m}^{-}\right)$$ (6)
  where *J*^+^ represents the index set for which the greater the better, and *J*^*-*^ represents the index set for which the smaller the better.
**Step 4:** Calculate the grey correlation coefficient between the *i*th alternative and positive-ideal alternative regarding the *j*th criterion. The procedure is presented as follows:
  $${\text{r}}_{ij}^{+}=\frac{\underset{i}{\text{min}}\;\underset{j}{\text{min}}\left|z_{j}^{+}-z_{ij}\right|+\rho \;\underset{i}{\text{max}}\;\underset{j}{\text{max}}\left|z_{j}^{+}-z_{ij}\right|}{\left|z_{j}^{+}-z_{ij}\right|+\rho \;\underset{i}{\text{max}}\;\underset{j}{\text{max}}\left|z_{j}^{+}-z_{ij}\right|}$$ (7)
  where *ρ*∈[0, 1] indicates the resolution factor. As a general rule, *ρ* = 0.5 [22].
  The grey correlation coefficient matrix regarding each alternative and positive-ideal solution is shown in Eq (8).
  $$R^{+}=\begin{array}{cc} & \begin{array}{ccccc} B_{1} & \cdots & B_{j} & \cdots & B_{m} \end{array}\\ \begin{array}{c} A_{1}\\ \vdots \\ A_{i}\\ \vdots \\ A_{n} \end{array} & \left[\begin{array}{ccccc} r_{11}^{+} & \cdots & r_{1j}^{+} & \cdots & r_{1m}^{+}\\ \vdots & \ddots & \vdots & \ddots & \vdots \\ r_{i1}^{+} & \cdots & r_{ij}^{+} & \cdots & r_{im}^{+}\\ \vdots & \ddots & \vdots & \ddots & \vdots \\ r_{n1}^{+} & \cdots & r_{nj}^{+} & \cdots & r_{nm}^{+} \end{array}\right] \end{array}$$ (8)
  The grey correlation degree between the *i*th alternative and the positive-ideal solution can be obtained according to Eq (9).
  $$R_{i}^{+}=\frac{1}{m}\sum_{j=1}^{m}r_{ij}^{+},\left(i=1,2,\cdots ,n\right)$$ (9)
**Step 5:** Obtain the grey correlation coefficient between the *i*th alternative and negative-ideal solution regarding the *j*th index in the same way of Step 4.
  $${\text{r}}_{ij}^{-}=\frac{\underset{i}{\text{min}}\;\underset{j}{\text{min}}\left|z_{j}^{-}-z_{ij}\right|+\rho \;\underset{i}{\text{max}}\;\underset{j}{\text{max}}\left|z_{j}^{-}-z_{ij}\right|}{\left|z_{j}^{-}-z_{ij}\right|+\rho \;\underset{i}{\text{max}}\;\underset{j}{\text{max}}\left|z_{j}^{-}-z_{ij}\right|}$$ (10)
  The grey correlation coefficient matrix regarding each alternative and negative-ideal solution is shown in Eq (11).
  $$R^{-}=\begin{array}{cc} & \begin{array}{ccccc} B_{1} & \cdots & B_{j} & \cdots & B_{m} \end{array}\\ \begin{array}{c} A_{1}\\ \vdots \\ A_{i}\\ \vdots \\ A_{n} \end{array} & \left[\begin{array}{ccccc} r_{11}^{-} & \cdots & r_{1j}^{-} & \cdots & r_{1m}^{-}\\ \vdots & \ddots & \vdots & \ddots & \vdots \\ r_{i1}^{-} & \cdots & r_{ij}^{-} & \cdots & r_{im}^{-}\\ \vdots & \ddots & \vdots & \ddots & \vdots \\ r_{n1}^{-} & \cdots & r_{nj}^{-} & \cdots & r_{nm}^{-} \end{array}\right] \end{array}$$ (11)
  The grey association degree between the *i*th alternative and the negative-ideal can be obtained according to Eq (12).
  $$R_{i}^{-}=\frac{1}{m}\sum_{j=1}^{m}r_{ij}^{-},\left(i=1,2,\cdots ,n\right)$$ (12)
**Step 6:** Obtain the separation measures. The separation of each alternative from the positive-ideal solution $D_{i}^{+}$ is calculated as
  $$D_{i}^{+}=\sqrt{\sum_{j=1}^{m}{\left[z_{ij}-z_{j}^{+}\right]}^{2}},\left(i=1,2,\cdots ,n\right)$$ (13)
  Similarly, the separation of each alternative from the negative-ideal solution $D_{i}^{-}$ is calculated as
  $$D_{i}^{-}=\sqrt{\sum_{j=1}^{m}{\left[z_{ij}-z_{j}^{-}\right]}^{2}},\left(i=1,2,\cdots ,n\right)$$ (14)
**Step 7:** Apply the dimensionless method to $R_{i}^{+}$, $R_{i}^{-}$, $D_{i}^{+}$ and $D_{i}^{-}$.
  $${\hat{M}}_{i}=\frac{M_{i}}{\underset{1\leq i\leq n}{\text{max}}M_{i}},\left(i=1,2,\cdots ,n\right)$$ (15)
  where *M*_*i*_ represents $R_{i}^{+}$, $R_{i}^{-}$, $D_{i}^{+}$ and $D_{i}^{-}$.
**Step 8:** Calculate the similarity closeness index and the distance closeness index. For GRA approach, $R_{i}^{+}$ represents the grey correlation degree between the *i*th alternative and the positive-ideal solution. The larger the value *R*_*i*_, the more similar the alternative *i* to the positive-ideal alternative, the better the alternative. Similarly, for TOPSIS approach, the larger the value *D*_*i*_, the larger the separation of alternative *i* from the negative-ideal alternative, the better the alternative.
  $$R_{i}=\frac{{\hat{R}}_{i}^{+}}{{\hat{R}}_{i}^{+}+{\hat{R}}_{i}^{-}},\left(i=1,2,\cdots ,n\right)$$ (16)
  $$D_{i}=\frac{{\hat{D}}_{i}^{-}}{{\hat{D}}_{i}^{+}+{\hat{D}}_{i}^{-}},\left(i=1,2,\cdots ,n\right)$$ (17)
**Step 9:** Construct the integrated closeness index. To avoid subjectivity and irrationality, nonlinear programming is applied to calculate the integrated closeness index *CS*_*i*_ based on the similarity closeness index and the distance closeness index. Assuming that these two indices have the same weight, the nonlinear programming model with constraints can be structured as follows:
  $$\{\begin{array}{l} \text{min}\sum_{i=1}^{n}\left[{\left(\xi_{i}\right)}^{2}+{\left(\delta_{i}\right)}^{2}\right]\\ \xi_{i}=CS_{i}-R_{i}\\ \delta_{i}=CS_{i}-D_{i}\\ \begin{array}{l} s.t.\text{min}(R_{i},D_{i})\leq CS_{i}\leq \text{max}(R_{i},D_{i})\\ \begin{array}{cc} & \;0<CS_{i}<1 \end{array} \end{array} \end{array},\left(i=1,2,\cdots ,n\right)$$
  Subsequently, a complex method and a penalty function method can be employed to address this nonlinear programming model [73–74]. Note that the larger the value *CS*_*i*_, the better the alternative.

### A hybrid MCDM method

**Fig 1:**
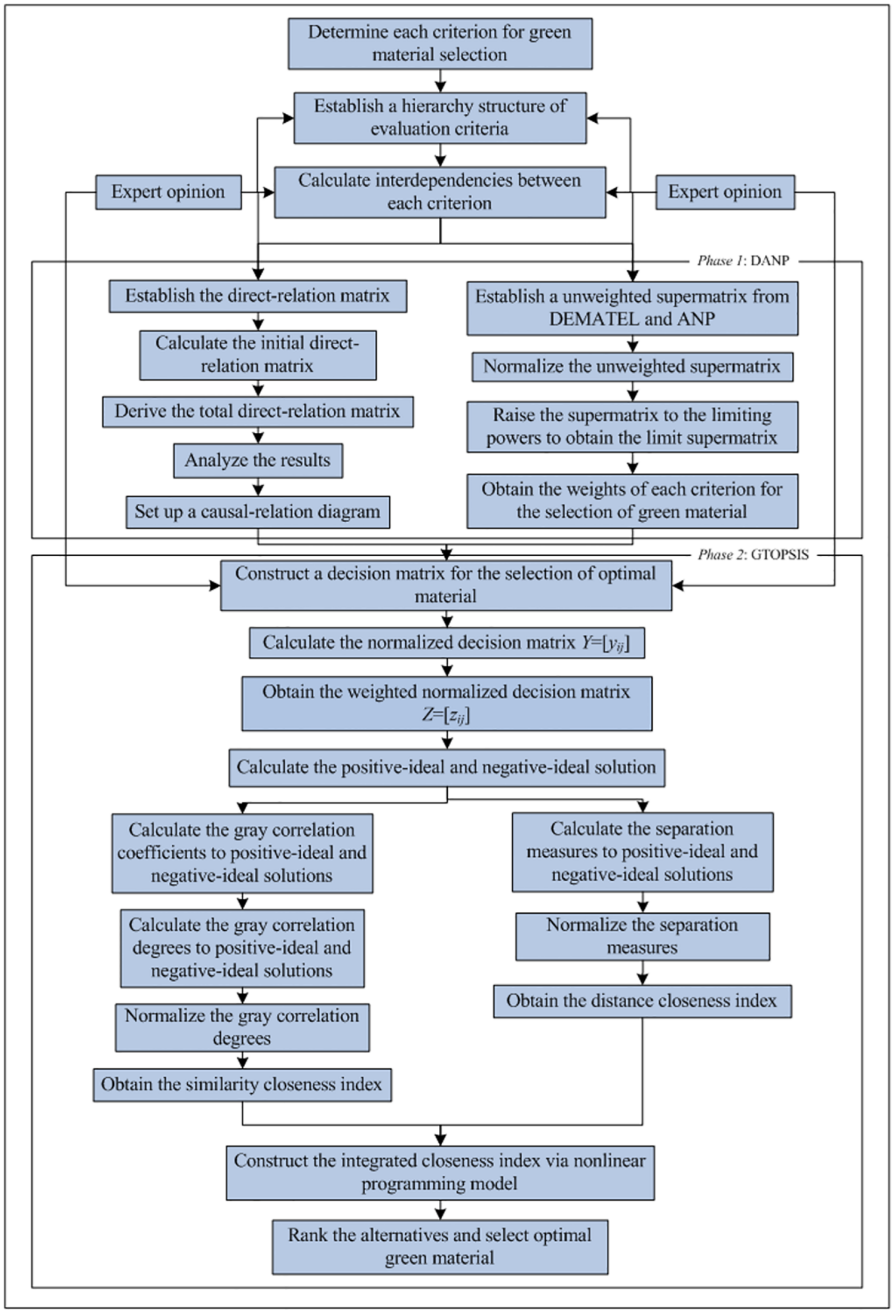
The flowchart of proposed novel hybrid method.

To select the optimal alternative more objectively and rationally, this work proposes a novel hybrid MCDM approach that combines DANP and G-TOPSIS. This method applies the quantitative analysis and weight allocation features of DANP and the comprehensive optimization ability of G-TOPSIS to avoid some of the shortcomings and deficiencies of each method alone. A detailed flowchart is shown in Fig 1.

**Phase 1:** Obtain the weights of each criterion for optimal green material selection via DANP.

**Fig 2:**
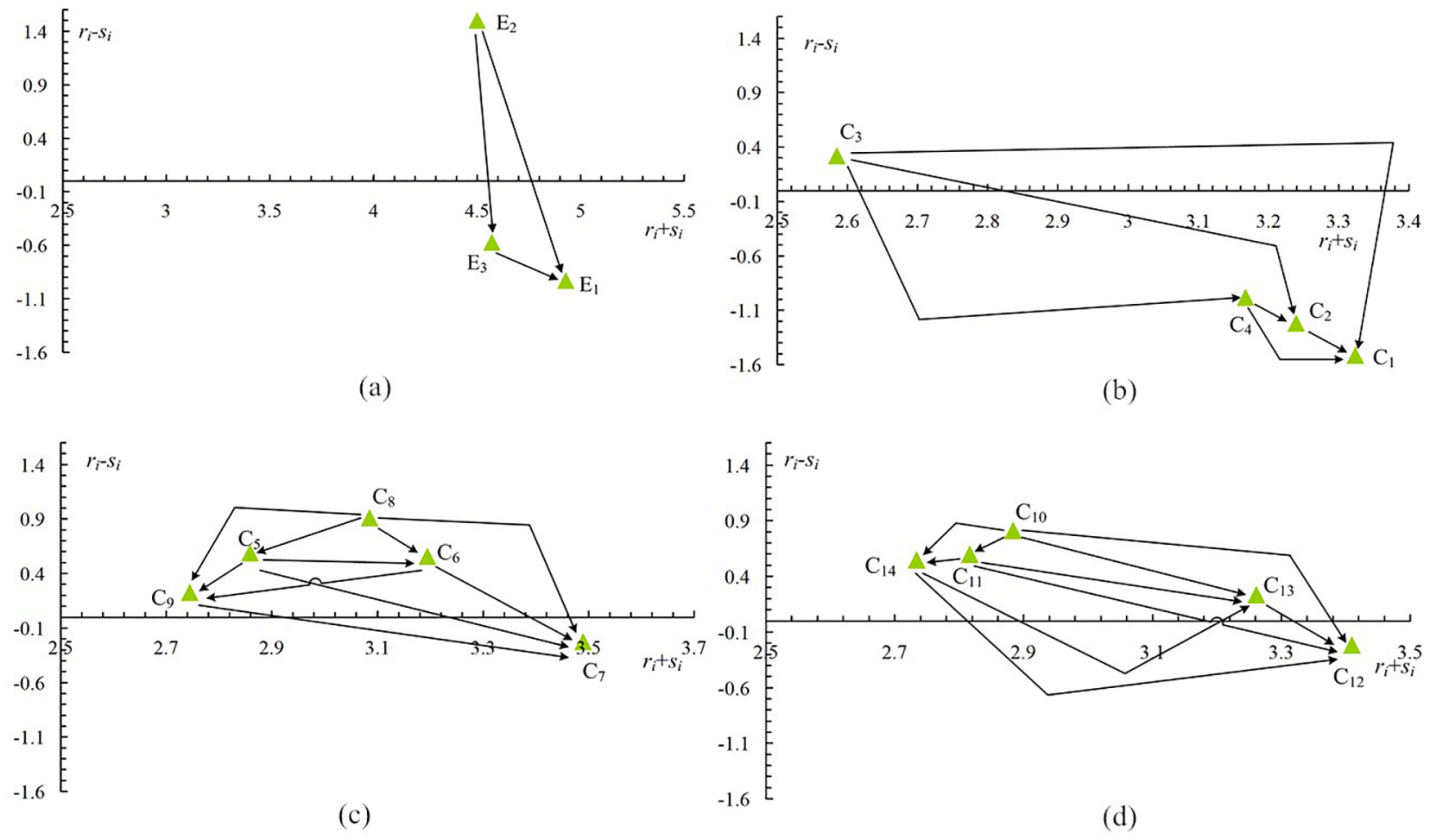
Causal influence diagrams for the dimensions and criteria.

The integrated DEMATEL and ANP process presented in Section 2.1 is applied to get the weights of each criterion while accounting for dependence and feedback. A hierarchical structure regarding the evaluation criteria is built, as shown in Fig 2. According to the classification of each criterion, DEMATEL is applied to analyze the interrelationship and influence of each criterion, and ANP is employed to calculate the weights of criteria.

**Phase 2:** Determine the final rank and select the optimal green material for certain product by G-TOPSIS.

The final rank of each alternative and optimal green material can be calculated via G-TOPSIS combining GRA and TOPSIS. Additionally, to avoid subjectivity and irrationality in the integration process, a nonlinear programming model with constraints is proposed to obtain the integrated closeness index *CS*_*i*_ based on the similarity closeness index *R*_*i*_ from GRA and the distance closeness index *D*_*i*_ from TOPSIS. Note that *CS*_*i*_ falls between 0 and 1. The larger the value of *CS*_*i*_, the better the performance of the material alternatives.

### Verification of the empirical case

An empirical case of rubbish bins is provided to demonstrate this integrated method, i.e., DANP and G-TOPSIS. In the following sections, the background, the hierarchical structure of criteria and the optimization processes will be discussed respectively.

### Background and data collection

As a common tool for daily life, the utilization and production of rubbish bins continue to increase; material selection is also an MCDM problem that must be treated with caution for sustainability. Currently, several commonly used material for rubbish bins are aluminum (Al), ABS plastic (ABS) and polyurethane (PU). In addition, production and recycling are closely related. While selecting a suitable material for production, it is necessary to choose corresponding product recovery and disposal strategies, i.e., recycling and remanufacturing (REM), recycling and incineration (INC), and recycling and landfill (LND). Thus, there are five material alternatives, i.e., Al-REM, ABS-INC, ABS-LND, PU-INC and PU-LND.

Raw data and related information can be gathered via experts from various fields, e.g., scholars of college and supervisors of enterprise, through questionnaire surveys. In this research, six experts, including two scholars who specialize in material selection, two supervisors from related companies with a good reputation, and two customers who have used these products for over three years, were interviewed to obtain the direct-relation matrix of each criterion and the decision matrix for the selection of optimal material. This investigation was conducted in August 2016. According to the statistical data from the six questionnaires, the inconsistent rate is 4.6% [75]. Thus, it can be summarized that the credibility is 95.40%, and additional questionnaires will not impact the optimization results.

### Hierarchical criteria of material selection

This large number of materials, coupled with the complex relationships between the different selection parameters, often make the selection of a materials for a given component a difficult task. The establishment of hierarchical criteria is a crucial step in green material selection, and it has great influence upon the accuracy and reliability of material alternatives evaluation. In selecting materials, designers and engineers have to take into account a large number of criteria. These criteria for material include social (e.g. operational life, esthetics, health and safety), technical (e.g., maintainability, resistance to decay, life expectancy), environmental (e.g., energy saving, potential for recycling and reuse, raw material extraction) and economic (initial cost, maintenance cost, disposal cost) [6, 76–78]. Physical properties have been applied in material selection, which plays an significant role in the assessment process for green material alternatives for real engineering processes [79–80]. However, there are rarely hierarchical structures that combining the physical properties with other important attributes, i.e., economic and environment, in the previous studies. In addition, there are no relatively accurate index weights for physical properties in the evaluation process of material selection.

Therefore, to discover and inherit more suitable sustainable properties/criteria, we reviewed the existing literature and interviewed experienced experts from colleges/enterprises. Thus, the hierarchical structure of criteria for their green material selection was built, as tabulated in Table 2. The structure includes three levels, i.e., goal, cluster and criterion. The goal level (G) is green material selection (G_1_). The cluster level (E) involves economic (E_1_), environment (E_2_), and physical (E_3_) properties. Economic properties include initial cost (C_1_), maintenance cost (C_2_), disposal cost (C_3_), and tax contribution (C_4_). Environment properties include energy saving (C_5_), potential for recycling and reuse (C_6_), raw material extraction (C_7_), usage of water (C_8_), and CO_2_ emission (C_9_). Physical properties include density (C_10_), rigidity (C_11_), tensile strength (C_12_), elongation at break (C_13_), and tensile modulus (C_14_). The attributes of each criterion are shown in Table 2. Note that rigidity (C_11_) and tensile modulus (C_14_) are fixed index (the closer the attribute value is to a fixed value *t*_*i*_, the better the attribute). The fixed value *t*_*i*_ is determined by the type of the product. To simplify the optimization process, we convert fixed index into cost index by calculating the evaluation value, i.e., the absolute value of the difference between the initial value with the fixed value. The smaller the evaluation value, the better the criterion.

**Table 2:** Hierarchical structure of criteria for material selection.

Goal level	Cluster level	Criterion level	Definitions	Attributes	References
**Green material selection (G**_**1**_**)**	**Economic (E**_**1**_**)**	Initial cost (C_1_)	The cost which is to be spent the material manufacturing	Cost	[5, 29, 51, 75, 78, 81–84]
		Maintenance cost (C_2_)	The cost which is to be spent for the maintenance in its effective lifetime	Cost	
		Disposal cost (C_3_)	The cost which is to be spent for end of life disposal of the material	Cost	
		Tax contribution (C_4_)	Tax involved and contributed by the material	Benefit	
	**Environment (E**_**2**_**)**	Energy saving (C_5_)	Net energy saved by the material	Benefit	[5, 29, 75, 82–87]
		Potential for recycling and reuse (C_6_)	Recycling and reuse capability of the material	Benefit	
		Raw material extraction (C_7_)	Limited extraction of the raw material for the manufacturing of the final material	Benefit	
		Usage of water (C_8_)	Usage of water involved in the life cycle of the material	Cost	
		CO_2_ emission (C_9_)	CO_2_ emission of the material in its useful life time	Cost	
	**Physical property (E**_**3**_**)**	Density (C_10_)	The estimated measure of content per functional and lexical units in total	Benefit	
		Rigidity (C_11_)	The capacity to resist a hard object pressed into its surface of local materials	Cost	
		Tensile strength (C_12_)	The ability to resist permanent deformation and destruction	Benefit	
		Elongation at break (C_13_)	The ratio of the original length and the displacement value when pull-off	Benefit	
		Tensile modulus (C_14_)	Elastic when stretched for materials	Cost	

### Weighting of criteria via DANP method

The calculation procedure is structured by combining DEMATEL with ANP (in Section 2.1 and Appendix A). As shown in Appendix A, the direct-relation matrix could be formulated from the responses of six experts (Due to space limitations, the averaged direct-relation matrix for criteria and dimensions are only given here as shown in Tables 3 and 4).

**Table 3:** The averaged direct-relation matrix for criteria.

	**C**_**1**_	**C**_**2**_	**C**_**3**_	**C**_**4**_	**C**_**5**_	**C**_**6**_	**C**_**7**_	**C**_**8**_	**C**_**9**_	**C**_**10**_	**C**_**11**_	**C**_**12**_	**C**_**13**_	**C**_**14**_
**C**_**1**_	0	1	1	1	1	1	1	1	1	1	1	1	1	1
**C**_**2**_	2	0	1	2	1	1	1	1	1	1	1	1	1	1
**C**_**3**_	2	2	0	2	1	1	2	1	1	1	1	1	1	1
**C**_**4**_	2	1	1	0	1	1	1	1	1	1	1	3	1	1
**C**_**5**_	3	4	3	4	0	1	3	1	1	1	1	1	3	1
**C**_**6**_	4	3	3	3	2	0	4	2	2	1	1	2	1	1
**C**_**7**_	3	3	2	3	1	1	0	1	1	2	3	3	1	1
**C**_**8**_	4	4	3	3	2	1	3	0	2	1	1	2	4	1
**C**_**9**_	3	3	1	4	2	1	2	1	0	1	1	2	1	1
**C**_**10**_	3	3	2	3	2	3	3	1	2	0	2	1	2	1
**C**_**11**_	2	3	1	1	1	3	2	2	2	2	0	3	1	2
**C**_**12**_	3	2	2	2	1	2	2	1	2	1	1	0	4	1
**C**_**13**_	3	3	1	1	1	2	3	2	1	1	1	4	0	3
**C**_**14**_	3	3	1	3	1	2	2	1	2	1	1	3	2	0

**Table 4:** The averaged direct-relation matrix for the dimensions.

	**E**_**1**_	**E**_**2**_	**E**_**3**_
**E**_**1**_	0	1	2
**E**_**2**_	3	0	2
**E**_**3**_	2	1	0

Based on the calculation steps of DANP, the final weight of each criterion can be acquired as follows: a) the normalized initial direct-relation matrix can be calculated using Eqs (18) and (19); b) the total direct-relation matrix is obtained through Eq (20); c) each row sum vector *r* and column sum vector *s* of the total direct-relation matrix *T* are separately produced, as shown in Eqs (21) and (22), and the results are shown in Tables 5 and 6; d) the causal influence diagram is established based on the *r*_*i*_ + *c*_*i*_ and *r*_*i*_—*c*_*i*_ values respectively. The causal influence diagrams of criteria and the dimensions are shown in Fig 2; e) the unweighted supermatrix can be developed through Eqs (23)–(28); f) the weighted supermatrix can be obtained according to Eqs (29)–(31); and g) the result of limiting the weighted supermatrix is shown in Table 6.

**Table 5:** Sum of the influences given and received regarding criteria.

	Criteria	*r*_*i*_	*c*_*i*_	*r*_*i*_ + *c*_*i*_	*r*_*i*_ − *c*_*i*_
**1**	Initial cost (C_1_)	0.9052	2.4182	3.3234	-1.5131
**2**	Maintenance cost (C_2_)	1.0104	2.2290	3.2393	-1.2186
**3**	Disposal cost (C_3_)	1.4522	1.1325	2.5847	0.3197
**4**	Tax contribution (C_4_)	1.0920	2.0752	3.1672	-0.9832
**5**	Energy saving (C_5_)	1.7247	1.1342	2.8588	0.5905
**6**	Potential for recycling and reuse (C_6_)	1.8758	1.3185	3.1944	0.5573
**7**	Raw material extraction (C_7_)	1.6316	1.8584	3.4900	-0.2268
**8**	Usage of water (C_8_)	1.9976	1.0878	3.0854	0.9097
**9**	CO_2_ emission (C_9_)	1.4857	1.2593	2.7450	0.2264
**10**	Density (C_10_)	1.8480	1.0359	2.8839	0.8121
**11**	Rigidity (C_11_)	1.7071	1.1092	2.8163	0.5979
**12**	Tensile strength (C_12_)	1.5968	1.8124	3.4093	-0.2156
**13**	Elongation at break (C_13_)	1.7483	1.5130	3.2613	0.2353
**14**	Tensile modulus (C_14_)	1.6407	1.0929	2.7336	0.5478

**Table 6:** Sum of the influences given and received regarding the dimensions.

	Criteria	*r*_*i*_	*c*_*i*_	*r*_*i*_ + *c*_*i*_	*r*_*i*_ − *c*_*i*_
**1**	Economic (E_1_)	2.0000	2.9286	4.9286	-0.9286
**2**	Environment (E_2_)	3.0000	1.5000	4.5000	1.5000
**3**	Physical property (E_3_)	2.0000	2.5714	4.5714	-0.5714

### Rank the material alternatives via G-TOPSIS method

The calculation procedure is structured by combining GRA with TOPSIS (in Section 2.2). By reviewing the related literature [5, 26] and investigations by experts, a decision matrix for five material alternatives, i.e., Al-REM, ABS-INC, ABS-LND, PU-INC and PU-LND, is constructed, as shown in Table 7. Note that the value of *t*_*i*_ is 55 for the rigidity (C_11_) and the value of *t*_*i*_ is 10 for the tensile modulus (C_14_).

**Table 7:** A decision matrix for five material alternatives.

	C_1_	C_2_	C_3_	C_4_	C_5_	C_6_	C_7_	C_8_	C_9_	C_10_ /g.cc^-1^	C_11_ /HBS	C_12_ /MPa	C_13_ /%	C_14_ /GPa
**Al-REM**	2	3	3	3	3	3	4	3	3	2.72	50	169	8	25.0
**ABS-INC**	2	3	2	3	2	3	3	2	2	1.34	100	90	2	7.9
**ABS-LND**	3	2	3	2	2	4	2	4	3	1.34	100	90	2	7.9
**PU-INC**	3	3	2	3	4	2	3	4	3	1.15	60	27	10	4.5
**PU-LND**	4	4	2	4	4	3	3	3	4	1.15	60	27	10	4.5
**Weight**	0.052	0.061	0.067	0.052	0.086	0.112	0.054	0.092	0.083	0.053	0.076	0.069	0.074	0.069

The ranking of five material alternatives can be calculated via G-TOPSIS method, as described in Section 2.2. The steps can be divided into the following five parts: a) from Steps 1 to 2, the normalized decision matrix *Z* combined with the weight vector of criteria ***ω*** is obtained; b) the positive-ideal and negative-ideal solutions can be calculated using Step 3; c) the grey correlation coefficient between the *i*th alternative and the positive-ideal alternative regarding the *j*th criterion is obtained via Step 4, and similarly, the grey correlation coefficient between the *i*th alternative and the negative-ideal solution regarding the *j*th index can be calculated using Step 5; d) the similarity closeness index and the distance closeness index are acquired according to Steps 6–8; and e) the integrated closeness index is gained using a nonlinear programming model with constraints as shown in Step 9, and the final rank can be obtained, with the ranking presented in Table 8.

**Table 8:** Ranking of five material alternatives.

	*R*_*i*_	Rank	*D*_*i*_	Rank	*CS*_*i*_	Rank
**Al-REM**	0.6228	1	0.7036	1	0.6632	1
**ABS-INC**	0.3965	5	0.3914	5	0.3940	5
**ABS-LND**	0.4762	4	0.4871	3	0.4816	3
**PU-INC**	0.4901	3	0.4609	4	0.4755	4
**PU-LND**	0.5472	2	0.5097	2	0.5285	2

## Analysis and discussion

### Comparison to existing methods

**Fig 3:**
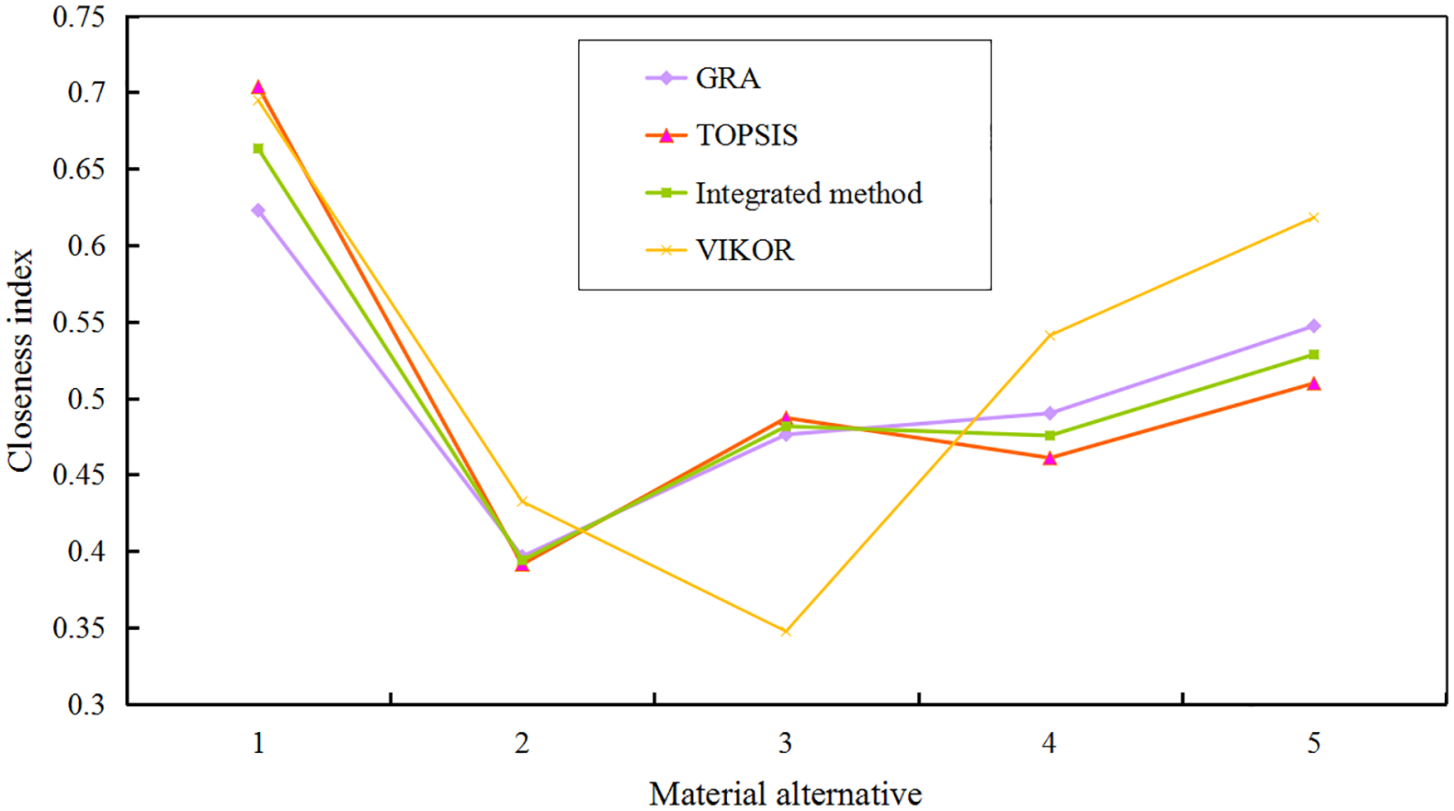
The closeness indices of the four methods.

To prove the feasibility and validity of the proposed method, GRA, TOPSIS and VIKOR [9, 88] were applied to compare their outcomes. Note that the same weights of criteria were applied in the calculation process of the four methods. The analysis is conducted on the basis of the same illustrative example. Based on Table 8, the integrated closeness indices of the four methods, i.e., GRA, TOPSIS, VIKOR and G-TOPSIS, can be figured in Fig 3.

From Fig 3, it can be summarized that the final ranks of material alternatives via the four methods are basically consistent. Thus, this proposed method, i.e., DANP and G-TOPSIS, is a reasonable and effective method to evaluate the performance of material alternatives and select the optimal green material. Subsequently, based on the results of the four methods, the first material alternative, that is Al-REM, is the optimal green material to produce rubbish bins. In addition, the ranks of the five material alternatives are different using the four methods. The causes of this phenomenon are summarized as follows: 1) the degree of information utilization is different in different information aggregation methods, and a large amount of information can be easily lost in the aggregation process; 2) the operating principium of TOPSIS is based on the distance from the positive-ideal solution and the negative-ideal solution; however, it does not consider the degree of similarity to the ideal solution; and 3) similarly. GRA only takes into account the degree of similarity to the ideal solution, thereby easily resulting in information loss. Therefore, we propose a hybrid MCDM approach combining DANP and G-TOPSIS to obtain the weight of each criterion and select the optimal green material logically and effectively. In addition, nonlinear programming is applied to make G-TOPSIS more reasonable.

### Sensitivity analysis

To monitor the robustness of the evaluation and selection for green materials, a sensitivity analysis that contains 19 experiments is conducted according to the weight change of each criterion. Table 9 presents the details of the experiment. For each condition, the integrated closeness indices of each material alternative are calculated. From Table 9, in the first 14 experiments, weights of each criterion are set as higher respectively, whereas the other criteria are set to be same. In experiment 15, the weights of criteria (C_1_–C_8_) = 0.125, whereas the other criteria weights are equal to zero. In experiment 16, the weights of criteria (C_9_–C_14_) = 0.167, whereas the other criteria weights are equal to zero. In experiment 17, the weights of all criteria of economic (C_1_–C_4_) = 0.25, whereas the other criteria weights are equal to zero. In experiment 18, the weights of all criteria of environment (C_5_–C_9_) = 0.2, whereas the other criteria weights are equal to zero. In experiment 19, the weights of all criteria of physical property (C_10_–C_14_) = 0.2, whereas the other criteria weights are equal to zero.

**Table 9:** The 19 experiments of sensitivity analysis.

Expt. No.	Weights	The integrated closeness index (*CS*_*i*_)					Rank
		Alternative 1	Alternative 2	Alternative 3	Alternative 4	Alternative 5	
1	*ω*_C1_ = 0.35, *ω*_C2-C14_ = 0.05	0.4679	0.3306	0.5272	0.5417	0.6377	5>4>3>1>2
2	*ω*_C2_ = 0.35, *ω*_C1, C3-C14_ = 0.05	0.6359	0.4896	0.3642	0.5363	0.6324	**1>5>4>2>3**
3	*ω*_C3_ = 0.35, *ω*_C1-C2, C4-C14_ = 0.05	0.7026	0.3411	0.5444	0.4006	0.4406	**1>3>5>4>2**
4	*ω*_C4_ = 0.35, *ω*_C1-C3, C5-C14_ = 0.05	0.5824	0.4429	0.3640	0.4894	0.6321	5>1>4>2>3
5	*ω*_C5_ = 0.35, *ω*_C1-C4, C6-C14_ = 0.05	0.5888	0.3315	0.3705	0.6101	0.6386	5>4>1>3>2
6	*ω*_C6_ = 0.35, *ω*_C1-C5, C7-C14_ = 0.05	0.5832	0.4436	0.5883	0.3746	0.5204	3>1>5>2>4
7	*ω*_C7_ = 0.35, *ω*_C1-C6, C8-C14_ = 0.05	0.7093	0.4327	0.3547	0.4790	0.5092	**1>5>4>2>3**
8	*ω*_C8_ = 0.35, *ω*_C1-C7, C9-C14_ = 0.05	0.6440	0.3332	0.5959	0.6120	0.5745	**1>4>3>5>2**
9	*ω*_C9_ = 0.35, *ω*_C1-C8, C10-C14_ = 0.05	0.6420	0.3315	0.5281	0.5426	0.6386	**1>5>4>3>2**
10	*ω*_C10_ = 0.35, *ω*_C1-C9, C11-C14_ = 0.05	0.7144	0.3302	0.3652	0.3549	0.3846	**1>5>3>4>2**
11	*ω*_C11_ = 0.35, *ω*_C1-C10, C12-C14_ = 0.05	0.7241	0.6152	0.6331	0.3236	0.3446	**1>3>2>5>4**
12	*ω*_C12_ = 0.35, *ω*_C1-C11, C13-C14_ = 0.05	0.7198	0.5451	0.5668	0.3353	0.3599	**1>3>2>5>4**
13	*ω*_C13_ = 0.35, *ω*_C1-C12, C4-C14_ = 0.05	0.6571	0.3089	0.3365	0.6489	0.6687	5>1>4>3>2
14	*ω*_C14_ = 0.35, *ω*_C1-C13_ = 0.05	0.7278	0.2997	0.3254	0.5751	0.5928	**1>5>4>3>2**
15	*ω*_C1-C8_ = 0.125, *ω*_C9-C14_ = 0	0.5311	0.3799	0.4606	0.5238	0.6139	5>1>4>3>2
16	*ω*_C1-C8_ = 0, *ω*_C9-C14_ = 0.167	0.7027	0.4133	0.4361	0.4239	0.4373	**1>5>3>4>2**
17	*ω*_C1-C4_ = 0.25, *ω*_C5-C14_ = 0	0.4613	0.3988	0.4116	0.4803	0.6039	5>4>1>3>2
18	*ω*_C5-C9_ = 0.2, *ω*_C1-C4, C10-C14_ = 0	0.6014	0.3746	0.5273	0.5691	0.6283	5>1>4>3>2
19	*ω*_C1-C9_ = 0, *ω*_C10-C14_ = 0.2	0.7117	0.4254	0.4254	0.4128	0.4128	**1>3 = 2>5 = 4**

**Fig 4:**
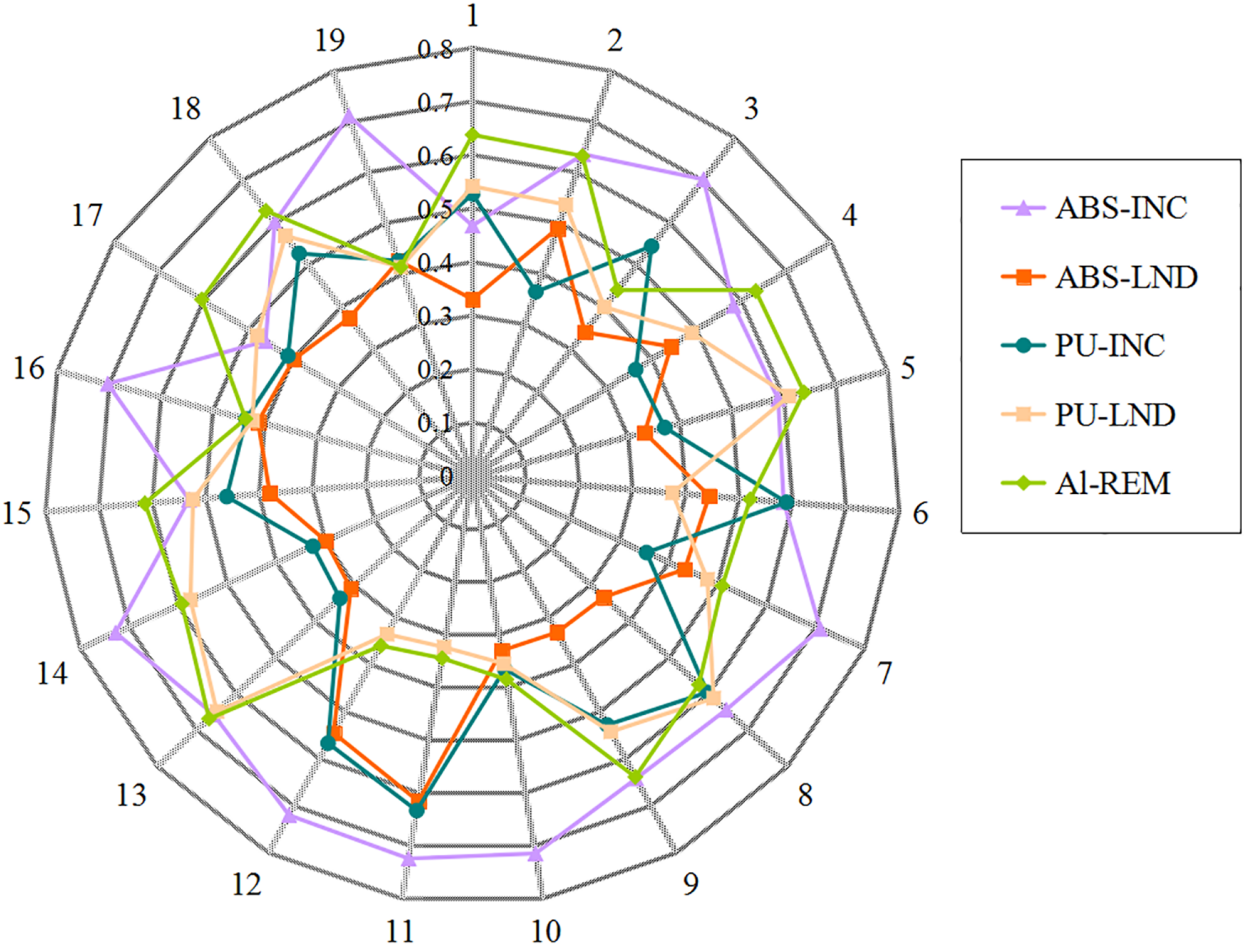
Sensitivity analysis.

As shown in Table 9, the changes in the final ranks of the five material alternatives when the weights of the criteria are changed can be figured in Fig 4. According to Table 9 and Fig 4, the following conclusions can be obtained as follows: 1) out of the 19 experiments, alternative 1, that is Al-REM, has the highest score in the 11 experiments, i.e., experiment numbers (2, 3, 7–12, 14, 16, 19); hence, the ranking of the solutions of the five material alternatives is relatively sensitive to the criteria weights; and 2) the final ranking of the alternatives changes greatly with the weight vary of each criterion. Therefore, obtaining the weight of each criterion reasonably and scientifically plays a significant role in the selection of the optimal green material.

## Discussion

By comparing the results from the three methods and the sensitivity analysis (as shown in Figs 3 and 4), it can be confirmed that this hybrid MCDM method is effective for the selection of the optimal choice from the material/design alternatives. Additionally, to illustrate that this method is better than the traditional methods, the significance of the present study can be summarized as follows:

From Table 6 and Fig 2. it can be seen that each cluster has feedback and dependence. In other words, the status of each cluster is different. In this paper, the degree that cluster 1 is affected by the other clusters (4.9286) is higher than the degrees for the others (4.5000, 4.5714). Thus, cluster 1 should occupy a greater proportion in the operation process. However, in the traditional method, e.g., AHP and ANP, it is assumed that each cluster has the same weight. The final weights of each cluster are either higher or lower than the realistic values. Therefore, this paper combines DEMATEL to improve the normalization of ANP in the unweighted supermatrix, and the results confirmed that it can be used to obtain the final weights of each cluster more reasonably. In addition, as shown in Table 8 and Fig 3, the final ranks from different methods are unstable, e.g., PU-INC is better than ABS-LND via DANP-GRA, in contrast, ABS-LND is better than PU-INC via DANP-TOPSIS. The reason for this difference is that each single method has its limitations, which will impact the final rank (the detailed limitation is summarized in the sub-section G-TOPSIS). Therefore, G-TOPSIS is proposed to rank the material alternatives effectively. To avoid subjectivity and irrationality, a nonlinear programming model with constraints is proposed to obtain the integrated closeness index based on the similarity closeness index from GRA and the distance closeness index from TOPSIS. In addition, a comparison and a sensitivity analysis are employed to confirm the accuracy and effectiveness.

The practical implication could be summarized from our study as follows: 1) by DANP, it can be seen that potential for recycling and reuse (C_6_) (0.112), usage of water (C_8_) (0.092), and energy saving (C_5_) (0.086) are found to have a large impact on the green material selection since these criteria carry relatively larger weights. Thus, their reasonable control can greatly contribute to a better design for engineers/designers. In addition, the results of the sensitivity analysis illustrate the importance of establishing a qualified group of experts/designers in the design evaluation. 2) The selection of the optimal material alternative is essential for the sustainable development of products. The main contribution of this work is the definition and development of an effective evaluation framework to guide managers to assess green material alternatives. To the best of our knowledge, no studies exist on devising a hybrid MCDM method that integrates DANP and G-TOPSIS to solve a green material selection problem for sustainable development. The results confirm that this method overcomes the one-sidedness of DANP-TOPSIS and DANP-GRA and makes the evaluation results more objective and realistic. In addition, the results of comparing with VIKOR confirmed that the final rank of this proposed approach is credible. Clearly, this study provides a more accurate, effective and systematic decision support tool for green material selection. In addition, this study can be useful for researchers to better understand the green material selection problem theoretically, as well as to organizations in designing/developing a better green design evaluation system.

## Conclusion

Sustainable development is a difficult and restrained task for all walks of life, e.g., the manufacturing industry and the environmental protection agency. It has also been a great concern to countries, especially developing countries. In addition, previous research studies have proposed many solutions and optimization methods, with determining how to select the optimal material being one of the key problems. In this paper, we proposed a hybrid MCDM approach that combines DANP and G-TOPSIS to evaluate the material alternatives and select the optimal material for sustainability. This method not only handles the complex interactions and interdependences among dimensions and criteria, but also provides a visible causal relationship diagram to obtain the weights of each criterion for material selection. In addition, it combines GRA and TOPSIS in view of the lack of a single MCDM method, and a nonlinear programming model with constraints is proposed to obtain the integrated closeness index to avoid subjectivity and irrationality during the integration process. An empirical application of rubbish bins was used to illustrate the proposed method. A sensitivity analysis and a comparison with existing methods were employed to validate the stability of the final results. The results of the research in this paper show the following:

1. the proposed approach combining DANP and G-TOPSIS is a reasonable and effective tool for green material selection based on the results of an empirical case, a comparison of methods, and a sensitivity analysis;
2. a suitable hierarchical structure of each criterion considering the economic, environment and physical properties was built for material selection, and
3. the weights of each criterion are obtained via DANP and a causal influence diagram for dimensions and criteria is built.

As future work, our studies will focus on three direction: 1) on the basis of this study, we will integrate other significant impact criteria, e.g., social, technical and interior environment characteristics factors in the hierarchical structure, and formulate a more complete index system; 2) this hybrid method could be applied to other fields. For example, green performance assessment and design alternative selection, and a computer-assisted design support system will be designed and applied in the assessment process; 3) by noting that the raw data from the experts have uncertain and imprecise features, uncertainty theory must be integrated in MCDM methods for further development [89–90].

## Appendix A

**Step 1**: Calculate the direct-relation matrix. The degree of direct impact that criterion *i* exerts on criterion *j*, which is denoted by *d*_*ij*_, can be formulated by several experts/engineers in this field based on assumed scales, i.e., "no influence (0)", "very low influence (1)", "low influence (2)", "high influence (3)" and "very high influence (4)". Subsequently, A direct-relation matrix *A* = [*a*_*ij*_]_*n×n*_ is produced through the mean of each same criterion in the various matrices of the experts/engineers.
**Step 2:** Establish the initial direct-relation matrix. The initial direct-relation matrix *D* = [*d*_*ij*_]_*n×n*_ can be derived through normalizing the matrix *A* as shown in Eqs (18) and (19).
  D=s×A (18)
  $$s=\text{min}\left[\frac{1}{{\text{max}}_{i}\sum_{j=1}^{n}\left|a_{ij}\right|},\frac{1}{{\text{max}}_{j}\sum_{i=1}^{n}\left|a_{ij}\right|}\right]$$ (19)
**Step 3:** Derive the total direct-relation matrix. Along the powers of *D*, e.g., *D*^2^, *D*^3^, …, *D*^*α*^, the indirect impact of each criterion is decreasing continuously. As *α* approaches infinity, then *D*^*α*^ = [0]_*n*×*n*_, where 0 ≤ *d*_*ij*_ <1, 0 < Σ_*i*_*d*_*ij*_ ≤ 1 and 0 < Σ_*j*_*d*_*ij*_ ≤ 1; at least one column sum Σ_*i*_*d*_*ij*_ or one row sum Σ_*j*_*d*_*ij*_ is equal to 1. Therefore, the total direct-relation matrix *T* = [*t*_*ij*_] _*n*×*n*_ can be obtained through Eq (20).
  $$T=D^{1}+D^{2}+\ldots +D^{\alpha }=D\left(I-D\right){\left(I-D\right)}^{-1}=D{\left(I-D\right)}^{-1}$$ (20)
  where lim_*α→*∞_*D*^*α*^ = [0]_*n*×*n*_.
**Step 4:** Analyze the results. Each row sum vector *r* and column sum vector *s* of total direct-relation matrix *T* are separately produced, as shown in Eqs (21) and (22), where *r*_*i*_ denotes the sum of total influences of criterion *i* on the other criteria. Similarly, *c*_*j*_ denotes the sum of total influences that criterion *j* has received from the other criteria. Additionally, (*r*_*i*_ + *c*_*i*_) and (*r*_*i*_—*c*_*i*_) should be calculated to analyze the results. (*r*_*i*_ + *c*_*i*_), as an index, can indicate the degree of the central role that criterion *i* plays in this problem when *i* = *j*. Regarding (*r*_*i*_—*c*_*i*_), if it's it is positive, criterion *i* affects other criteria; in contrast, criterion *i* is impacted by other criteria [32, 36].
  $$r={\left(r_{i}\right)}_{n\times 1}={\left[\sum_{j=1}^{n}t_{ij}\right]}_{n\times 1}$$ (21)
  $$c={\left(c_{j}\right)}_{n\times 1}={\left(c_{j}\right)}_{1\times n}^{\prime }={\left[\sum_{i=1}^{n}t_{ij}\right]}_{1\times n}^{\prime }$$ (22)
**Step 5:** Establish a causal-relation diagram. By mapping the data set of (*r*_*i*_ + *c*_*i*_, *r*_*i*_*—c*_*i*_), a causal-relation diagram can be structured to provide an effective method to determine how the preferred values in each dimension/cluster and criterion can be improved.
**Step 6:** Calculate the unweighted supermatrix. Two different total direct-relation matrices are then obtained from DEMATEL, i.e., $T_{C}={\left[t_{C}^{ij}\right]}_{n\times n}$ which pertains to *n* criteria and $T_{D}={\left[t_{D}^{ij}\right]}_{m\times m}$ which is devoted to *m* dimensions/clusters from *T*_*C*_ as shown in Eq (23).
  $$T_{C}=\begin{array}{ll} & \begin{array}{lllll} D_{1} & \cdots & D_{j} & \cdots & D_{m}\\ c_{11}\ldots c_{1n_{1}} & & c_{j1}\ldots c_{jn_{j}} & & c_{m1}\ldots c_{mn_{m}} \end{array}\\ \begin{array}{ll} D_{1} & \begin{array}{c} c_{11}\\ \vdots \\ c_{1n_{1}} \end{array}\\ \vdots & \\ D_{j} & \begin{array}{c} c_{i1}\\ \vdots \\ c_{in_{i}} \end{array}\\ \vdots & \\ D_{m} & \begin{array}{c} c_{m1}\\ \vdots \\ c_{mn_{m}} \end{array} \end{array} & \left[\begin{array}{ccccc} T_{C}^{11} & \begin{array}{ccc} & \cdots & \end{array} & T_{C}^{1j} & \begin{array}{ccc} & \cdots & \end{array} & T_{C}^{1m}\\ \begin{array}{c} \vdots \end{array} & \ddots & \vdots & \ddots & \vdots \\ T_{C}^{i1} & \cdots & T_{C}^{ij} & \cdots & T_{C}^{im}\\ & \ddots & \vdots & \ddots & \vdots \\ T_{C}^{m1} & \cdots & T_{C}^{mj} & \cdots & T_{C}^{mm} \end{array}\right] \end{array}$$ (23)
  In addition, a new matrix $T_{C}^{\delta }$ will be established by normalizing the total direct-relation matrix *T*_*C*_, as shown in Eqs (24) and (25).
  $$T_{C}^{\delta }=\begin{array}{ll} & \begin{array}{lllll} D_{1} & \cdots & D_{j} & \cdots & D_{m}\\ c_{11}\ldots c_{1n_{1}} & & c_{j1}\ldots c_{jn_{j}} & & c_{m1}\ldots c_{mn_{m}} \end{array}\\ \begin{array}{ll} D_{1} & \begin{array}{c} c_{11}\\ \vdots \\ c_{1n_{1}} \end{array}\\ \vdots & \\ D_{j} & \begin{array}{c} c_{i1}\\ \vdots \\ c_{in_{i}} \end{array}\\ \vdots & \\ D_{m} & \begin{array}{c} c_{m1}\\ \vdots \\ c_{mn_{m}} \end{array} \end{array} & \left[\begin{array}{ccccc} T_{C}^{\delta 11} & \begin{array}{ccc} & \cdots & \end{array} & T_{C}^{\delta 1j} & \begin{array}{ccc} & \cdots & \end{array} & T_{C}^{\delta 1m}\\ \begin{array}{c} \vdots \end{array} & \ddots & \vdots & \ddots & \vdots \\ T_{C}^{\delta i1} & \cdots & T_{C}^{\delta ij} & \cdots & T_{C}^{\delta im}\\ & \ddots & \vdots & \ddots & \vdots \\ T_{C}^{\delta m1} & \cdots & T_{C}^{\delta mj} & \cdots & T_{C}^{\delta mm} \end{array}\right] \end{array}$$ (24)
  An explanation for the normalization $T_{C}^{\delta 11}$ is explained in detail which is shown as Eqs (25) and (26). Similarly, other $T_{C}^{\delta ij}$ values can be obtained in the same manner.
  $$T_{C}^{\delta 11}={\left[t_{cij}^{\delta 11}\right]}_{m_{1}\times m_{1}}=\left[\begin{array}{ccccc} t_{c11}^{11}/d_{c1}^{11} & \cdots & t_{c1j}^{11}/d_{c1}^{11} & \cdots & t_{c1m_{1}}^{11}/d_{c1}^{11}\\ \vdots & \ddots & \vdots & \ddots & \vdots \\ t_{ci1}^{11}/d_{ci}^{11} & \cdots & t_{cij}^{11}/d_{ci}^{11} & \cdots & t_{cim_{1}}^{11}/d_{ci}^{11}\\ \vdots & \ddots & \vdots & \ddots & \vdots \\ t_{cm_{1}1}^{11}/d_{cm_{1}}^{11} & \cdots & t_{cm_{1}j}^{11}/d_{cm_{1}}^{11} & \cdots & t_{cm_{1}m_{1}}^{11}/d_{cm_{1}}^{11} \end{array}\right]$$ (25)
  $$d_{ci}^{11}=\sum_{j=1}^{m_{1}}t_{ij}^{11},i=1,2,\ldots ,m_{1}$$ (26)
  Let the total direct-relation matrix match and fill into the interdependence clusters. An unweighted supermatrix *W* can be obtained based on transposing the normalized total direct-relation matrix $T_{C}^{\delta }$, as shown in Eq (27).
  $$W={\left(T_{C}^{\delta }\right)}^{\prime }=\begin{array}{ll} & \begin{array}{lllll} D_{1} & \cdots & D_{j} & \cdots & D_{m}\\ c_{11}\ldots c_{1n_{1}} & & c_{j1}\ldots c_{jn_{j}} & & c_{m1}\ldots c_{mn_{m}} \end{array}\\ \begin{array}{ll} D_{1} & \begin{array}{c} c_{11}\\ \vdots \\ c_{1n_{1}} \end{array}\\ \vdots & \\ D_{j} & \begin{array}{c} c_{i1}\\ \vdots \\ c_{in_{i}} \end{array}\\ \vdots & \\ D_{m} & \begin{array}{c} c_{m1}\\ \vdots \\ c_{mn_{m}} \end{array} \end{array} & \left[\begin{array}{ccccc} W^{11} & \begin{array}{ccc} & \cdots & \end{array} & W^{i1} & \begin{array}{ccc} & \cdots & \end{array} & W^{n1}\\ \begin{array}{c} \vdots \end{array} & \ddots & \vdots & \ddots & \vdots \\ W^{1j} & \cdots & W^{ij} & \cdots & W^{nj}\\ & \ddots & \vdots & \ddots & \vdots \\ W^{1n} & \cdots & W^{in} & \cdots & W^{nn} \end{array}\right] \end{array}$$ (27)
  An explanation for the *W*^11^ is explained in detail which is shown as Eq (28). Similarly, other *W*^*ij*^ values can be obtained in the same manner.
  $$W^{11}=\begin{array}{cc} & \begin{array}{ccccc} c_{11} & \cdots & c_{1i} & \cdots & c_{1m_{1}} \end{array}\\ \begin{array}{c} c_{11}\\ \vdots \\ c_{1j}\\ \vdots \\ c_{1m_{1}} \end{array} & \left[\begin{array}{ccccc} t_{c11}^{\delta 11} & \cdots & t_{ci1}^{\delta 11} & \cdots & t_{cm_{1}1}^{\delta 11}\\ \vdots & \ddots & \vdots & \ddots & \vdots \\ t_{c1j}^{\delta 11} & \cdots & t_{cij}^{\delta 11} & \cdots & t_{cm_{1}j}^{\delta 11}\\ \vdots & \ddots & \vdots & \ddots & \vdots \\ t_{c1m_{1}}^{\delta 11} & \cdots & t_{cim_{1}}^{\delta 11} & \cdots & t_{cm_{1}m_{1}}^{\delta 11} \end{array}\right] \end{array}$$ (28)
**Step 7:** Calculate the weighted supermatrix. Each column will be summed for normalization as Eq (29).
  $$T_{D}=\left[\begin{array}{ccccc} t_{D}^{11} & \cdots & t_{D}^{1j} & \cdots & t_{D}^{1n}\\ \vdots & \ddots & \vdots & \ddots & \vdots \\ t_{D}^{i1} & \cdots & t_{D}^{ij} & \cdots & t_{D}^{in}\\ \vdots & \ddots & \vdots & \ddots & \vdots \\ t_{D}^{n1} & \cdots & t_{D}^{nj} & \cdots & t_{D}^{nn} \end{array}\right]$$ (29)
  A new matrix $T_{D}^{\delta }$ can be established by normalizing the total direct-relation matrix *T*_*D*_, as shown in Eq (30).
  $$T_{D}^{\delta }=[t_{D}^{\delta ij}]=\left[\begin{array}{ccccc} t_{D}^{11}/d_{1} & \cdots & t_{D}^{1j}/d_{1} & \cdots & t_{D}^{1n}/d_{1}\\ \vdots & \ddots & \vdots & \ddots & \vdots \\ t_{D}^{i1}/d_{i} & \cdots & t_{D}^{ij}/d_{i} & \cdots & t_{D}^{in}/d_{i}\\ \vdots & \ddots & \vdots & \ddots & \vdots \\ t_{D}^{n1}/d_{n} & \cdots & t_{D}^{nj}/d_{n} & \cdots & t_{D}^{nn}/d_{n} \end{array}\right]$$ (30)
  To obtain the weighted supermatrix, the normalized total direct-relation matrix $T_{D}^{\delta }$ must be multiplied as shown in Eq (31).
  $$W^{\delta }=T_{D}^{\delta }\times W=\left[\begin{array}{ccccc} t_{D}^{\delta 11}\times W^{11} & \cdots & t_{D}^{\delta i1}\times W^{i1} & \cdots & t_{D}^{\delta n1}\times W^{n1}\\ \vdots & \ddots & \vdots & \ddots & \vdots \\ t_{D}^{\delta 1j}\times W^{1j} & \cdots & t_{D}^{\delta ij}\times W^{ij} & \cdots & t_{D}^{\delta nj}\times W^{nj}\\ \vdots & \ddots & \vdots & \ddots & \vdots \\ t_{D}^{\delta 1n}\times W^{1n} & \cdots & t_{D}^{\delta in}\times W^{in} & \cdots & t_{D}^{\delta nn}\times W^{nn} \end{array}\right]$$ (31)
**Step 8:** Limit the weighted supermatrix. Limit the weighted supermatrix by raising it to a sufficiently large power *k* until the supermatrix converges and becomes a long-term stable supermatrix to obtain the global priority vector ***ω***.
